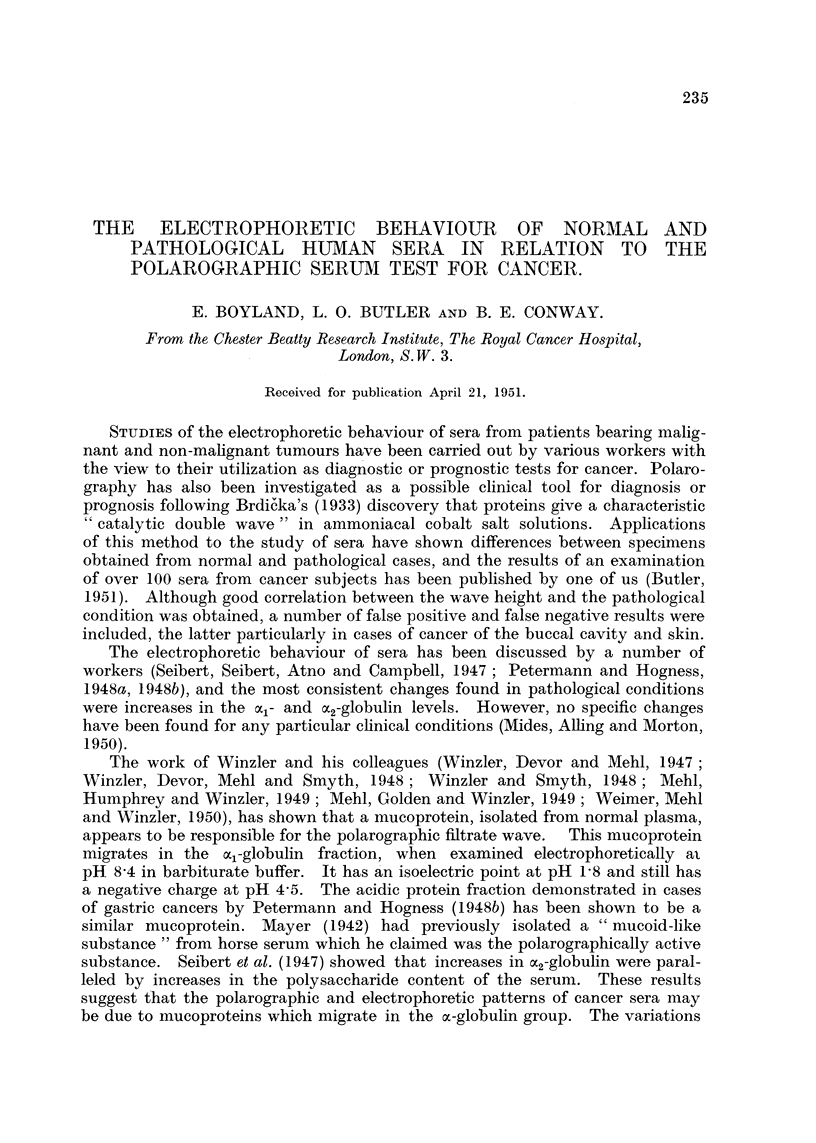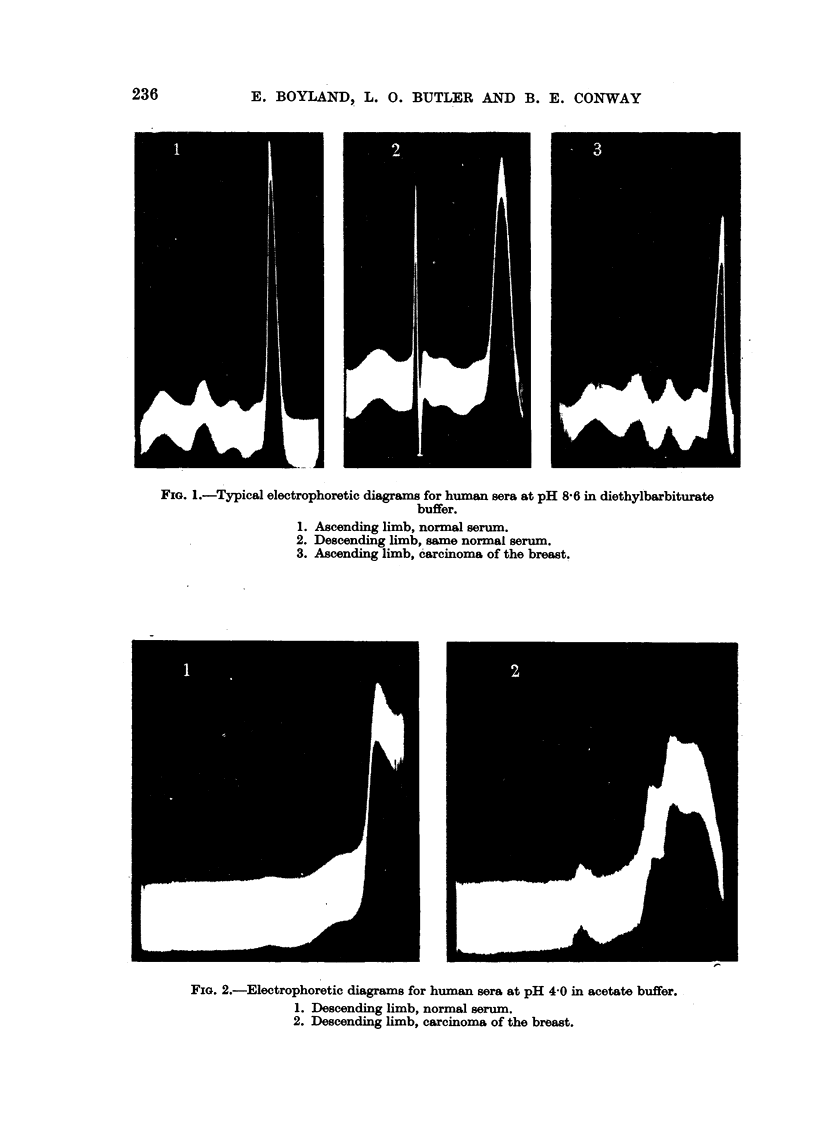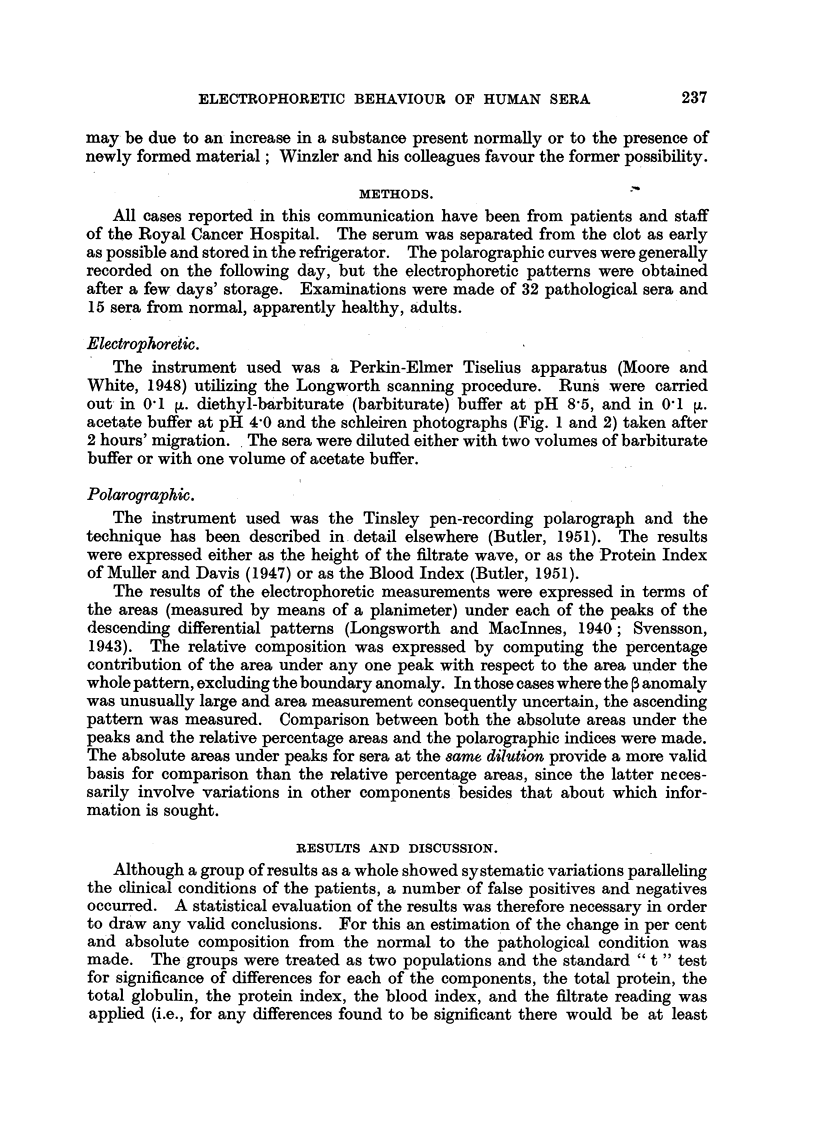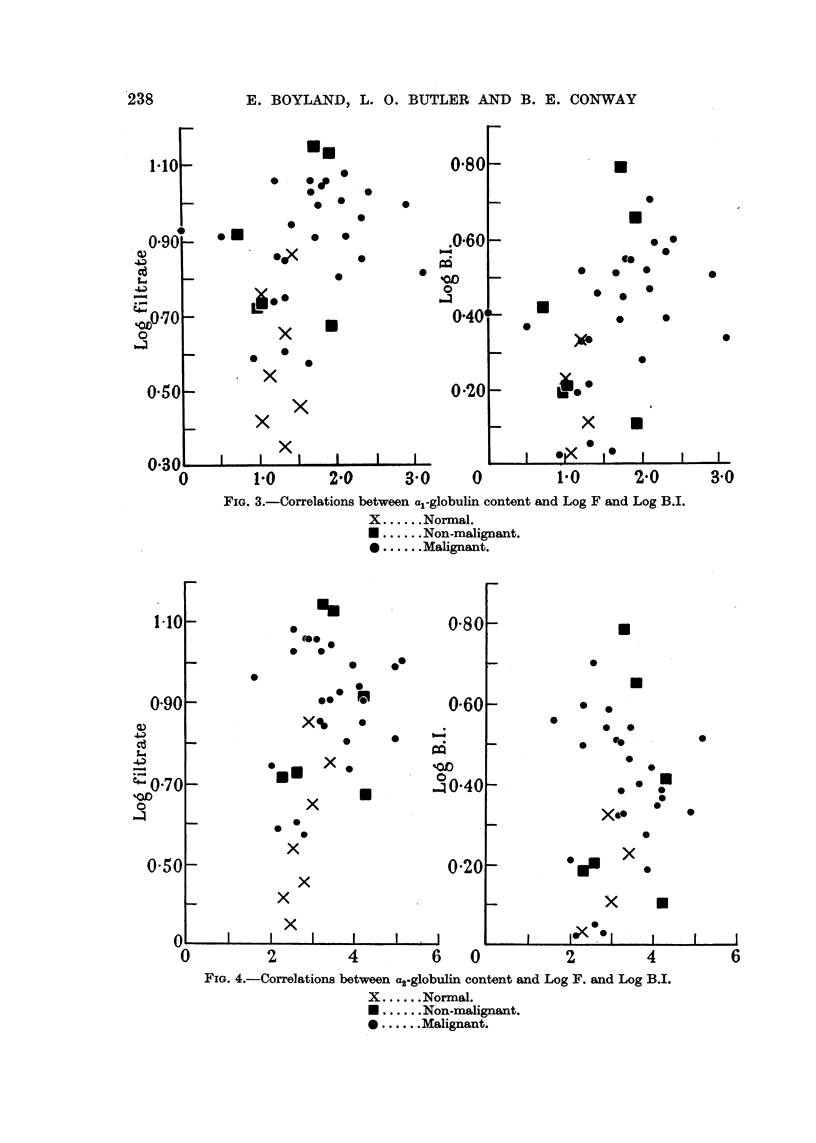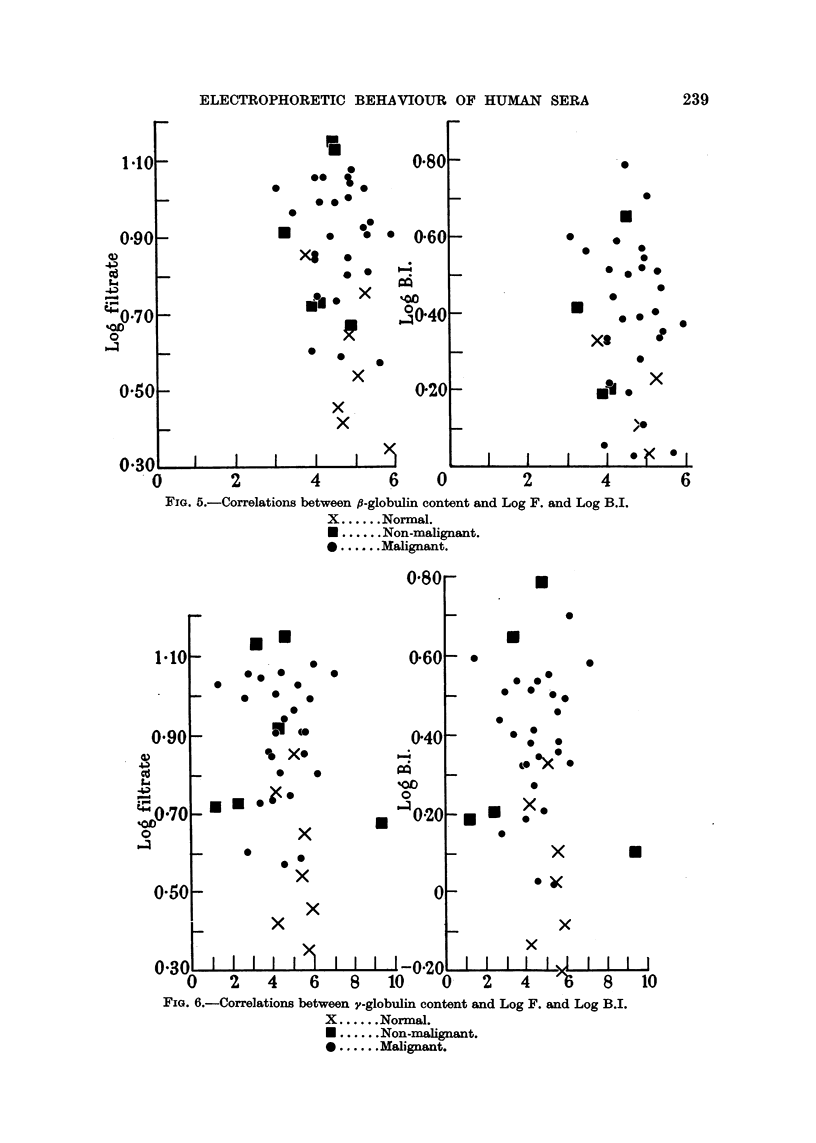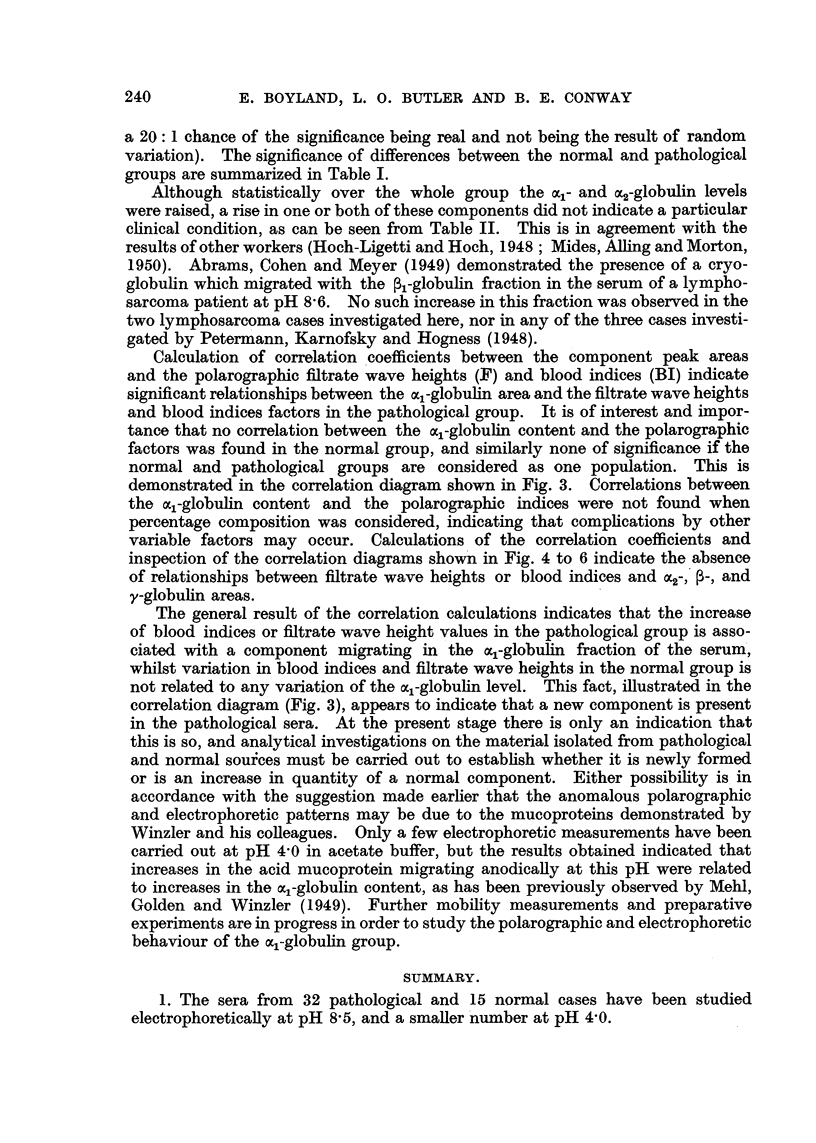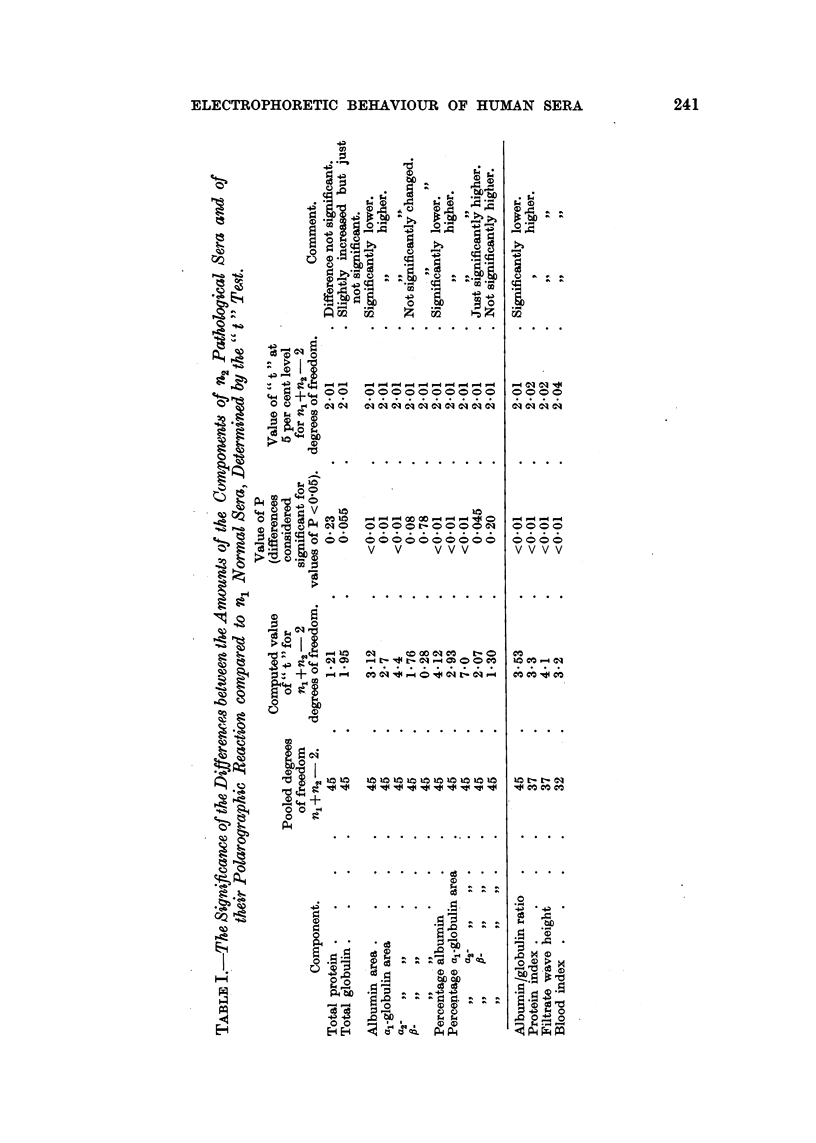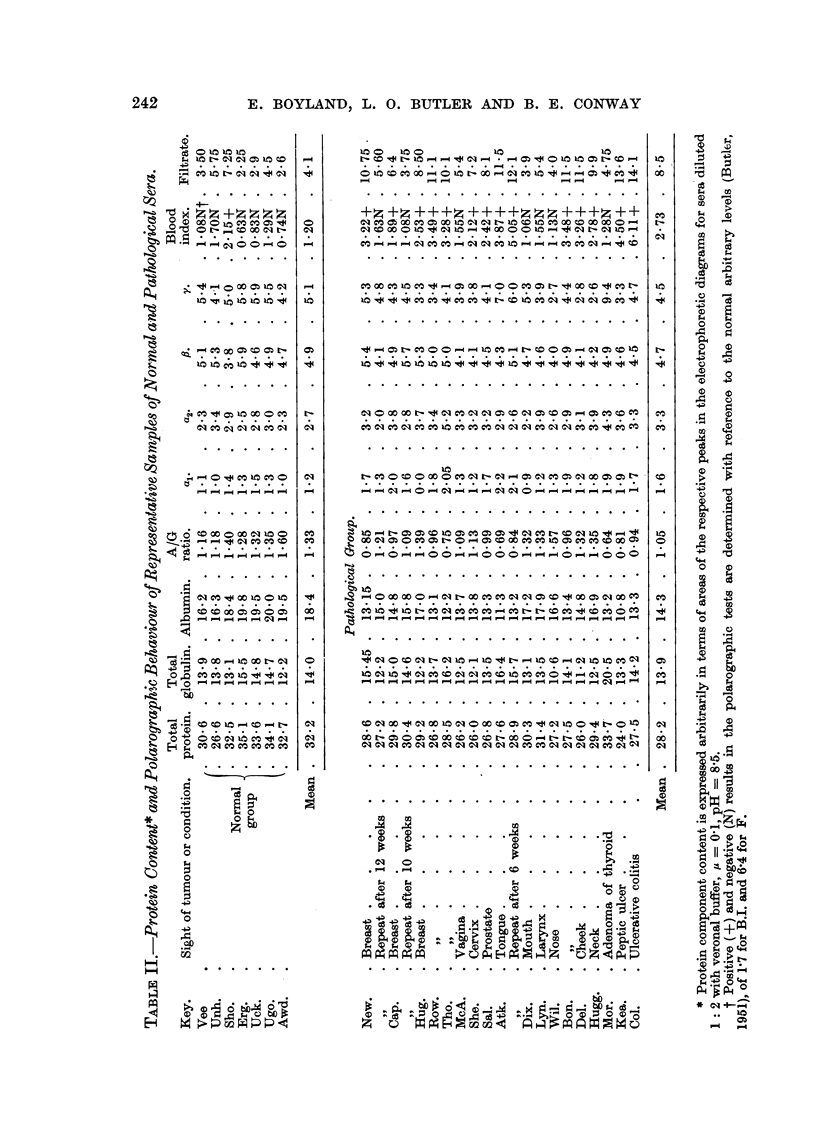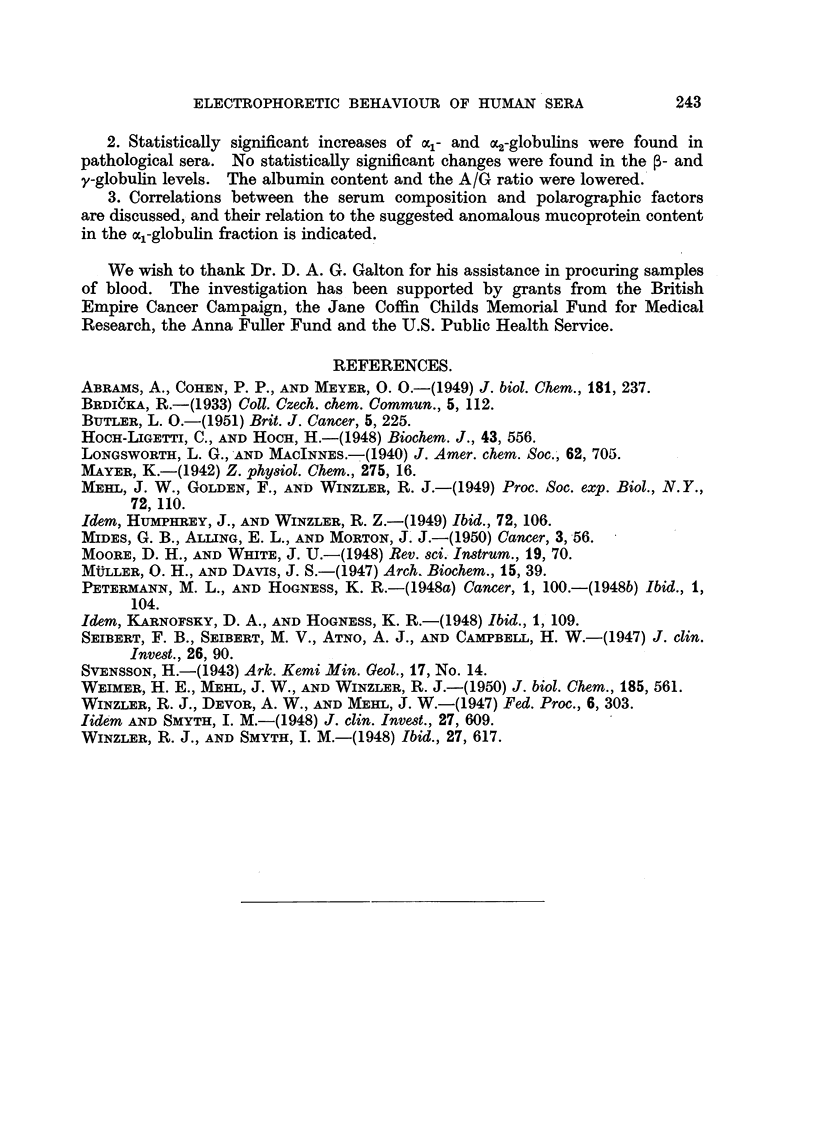# The Electrophoretic Behaviour of Normal and Pathological Human Sera in Relation to the Polarographic Serum Test for Cancer

**DOI:** 10.1038/bjc.1951.24

**Published:** 1951-06

**Authors:** E. Boyland, L. O. Butler, B. E. Conway

## Abstract

**Images:**


### THE ELECTROPHORETIC BEHAVIOUR OF NORMAL AND PATHOLOGICAL HUMAN SERA IN RELATION TO THE POLAROGRAPHIC SERUM TEST FOR CANCER.

E. BOYLAND, L. O. BUTLER AND B. E. CONWAY.

*From the Chester Beatty Research Institute, The Royal Cancer Hospital, London, S.W. 3.*

Received for publication April 21, 1951.

STUDIES of the electrophoretic behaviour of sera from patients bearing malignant and non-malignant tumours have been carried out by various workers with the view to their utilization as diagnostic or prognostic tests for cancer. Polarography has also been investigated as a possible clinical tool for diagnosis or prognosis following Brdička's (1933) discovery that proteins give a characteristic " catalytic double wave " in ammoniacal cobalt salt solutions. Applications of this method to the study of sera have shown differences between specimens obtained from normal and pathological cases, and the results of an examination of over 100 sera from cancer subjects has been published by one of us (Butler, 1951). Although good correlation between the wave height and the pathological condition was obtained, a number of false positive and false negative results were included, the latter particularly in cases of cancer of the buccal cavity and skin.

The electrophoretic behaviour of sera has been discussed by a number of workers (Seibert, Seibert, Atno and Campbell, 1947 ; Petermann and Hogness, 1948*a*, 1948*b*), and the most consistent changes found in pathological conditions were increases in the $\alpha_1$- and $\alpha_2$-globulin levels. However, no specific changes have been found for any particular clinical conditions (Mides, Alling and Morton, 1950).

The work of Winzler and his colleagues (Winzler, Devor and Mehl, 1947 ; Winzler, Devor, Mehl and Smyth, 1948 ; Winzler and Smyth, 1948 ; Mehl, Humphrey and Winzler, 1949 ; Mehl, Golden and Winzler, 1949 ; Weimer, Mehl and Winzler, 1950), has shown that a mucoprotein, isolated from normal plasma, appears to be responsible for the polarographic filtrate wave. This mucoprotein migrates in the $\alpha_1$-globulin fraction, when examined electrophoretically aι pH 8·4 in barbiturate buffer. It has an isoelectric point at pH 1·8 and still has a negative charge at pH 4·5. The acidic protein fraction demonstrated in cases of gastric cancers by Petermann and Hogness (1948*b*) has been shown to be a similar mucoprotein. Mayer (1942) had previously isolated a " mucoid-like substance " from horse serum which he claimed was the polarographically active substance. Seibert *et al.* (1947) showed that increases in $\alpha_2$-globulin were paralleled by increases in the polysaccharide content of the serum. These results suggest that the polarographic and electrophoretic patterns of cancer sera may be due to mucoproteins which migrate in the $\alpha$-globulin group. The variations

**Fig. 1.**—Typical electrophoretic diagrams for human sera at pH 8·6 in diethylbarbiturate buffer.

1. Ascending limb, normal serum.
2. Descending limb, same normal serum.
3. Ascending limb, carcinoma of the breast.

**Fig. 2.**—Electrophoretic diagrams for human sera at pH 4·0 in acetate buffer.

1. Descending limb, normal serum.
2. Descending limb, carcinoma of the breast.

may be due to an increase in a substance present normally or to the presence of newly formed material; Winzler and his colleagues favour the former possibility.

### METHODS.

All cases reported in this communication have been from patients and staff of the Royal Cancer Hospital. The serum was separated from the clot as early as possible and stored in the refrigerator. The polarographic curves were generally recorded on the following day, but the electrophoretic patterns were obtained after a few days' storage. Examinations were made of 32 pathological sera and 15 sera from normal, apparently healthy, adults.

*Electrophoretic.*

The instrument used was a Perkin-Elmer Tiselius apparatus (Moore and White, 1948) utilizing the Longworth scanning procedure. Runs were carried out in 0·1 μ. diethyl-barbiturate (barbiturate) buffer at pH 8·5, and in 0·1 μ. acetate buffer at pH 4·0 and the schleiren photographs (Fig. 1 and 2) taken after 2 hours' migration. The sera were diluted either with two volumes of barbiturate buffer or with one volume of acetate buffer.

*Polarographic.*

The instrument used was the Tinsley pen-recording polarograph and the technique has been described in detail elsewhere (Butler, 1951). The results were expressed either as the height of the filtrate wave, or as the Protein Index of Muller and Davis (1947) or as the Blood Index (Butler, 1951).

The results of the electrophoretic measurements were expressed in terms of the areas (measured by means of a planimeter) under each of the peaks of the descending differential patterns (Longsworth and MacInnes, 1940; Svensson, 1943). The relative composition was expressed by computing the percentage contribution of the area under any one peak with respect to the area under the whole pattern, excluding the boundary anomaly. In those cases where the β anomaly was unusually large and area measurement consequently uncertain, the ascending pattern was measured. Comparison between both the absolute areas under the peaks and the relative percentage areas and the polarographic indices were made. The absolute areas under peaks for sera at the *same dilution* provide a more valid basis for comparison than the relative percentage areas, since the latter necessarily involve variations in other components besides that about which information is sought.

### RESULTS AND DISCUSSION.

Although a group of results as a whole showed systematic variations paralleling the clinical conditions of the patients, a number of false positives and negatives occurred. A statistical evaluation of the results was therefore necessary in order to draw any valid conclusions. For this an estimation of the change in per cent and absolute composition from the normal to the pathological condition was made. The groups were treated as two populations and the standard " t " test for significance of differences for each of the components, the total protein, the total globulin, the protein index, the blood index, and the filtrate reading was applied (i.e., for any differences found to be significant there would be at least

Fig. 3.—Correlations between $\alpha_1$-globulin content and Log F and Log B.I.

X......Normal.
■......Non-malignant.
●......Malignant.

Fig. 4.—Correlations between $\alpha_2$-globulin content and Log F. and Log B.I.

X......Normal.
■......Non-malignant.
●......Malignant.

FIG. 5.—Correlations between β-globulin content and Log F. and Log B.I.
X......Normal.
■......Non-malignant.
●......Malignant.

FIG. 6.—Correlations between γ-globulin content and Log F. and Log B.I.
X......Normal.
■......Non-malignant.
●......Malignant.

a 20 : 1 chance of the significance being real and not being the result of random variation). The significance of differences between the normal and pathological groups are summarized in Table I.

Although statistically over the whole group the $\alpha_1$- and $\alpha_2$-globulin levels were raised, a rise in one or both of these components did not indicate a particular clinical condition, as can be seen from Table II. This is in agreement with the results of other workers (Hoch-Ligetti and Hoch, 1948 ; Mides, Alling and Morton, 1950). Abrams, Cohen and Meyer (1949) demonstrated the presence of a cryoglobulin which migrated with the $\beta_1$-globulin fraction in the serum of a lymphosarcoma patient at pH 8·6. No such increase in this fraction was observed in the two lymphosarcoma cases investigated here, nor in any of the three cases investigated by Petermann, Karnofsky and Hogness (1948).

Calculation of correlation coefficients between the component peak areas and the polarographic filtrate wave heights (F) and blood indices (BI) indicate significant relationships between the $\alpha_1$-globulin area and the filtrate wave heights and blood indices factors in the pathological group. It is of interest and importance that no correlation between the $\alpha_1$-globulin content and the polarographic factors was found in the normal group, and similarly none of significance if the normal and pathological groups are considered as one population. This is demonstrated in the correlation diagram shown in Fig. 3. Correlations between the $\alpha_1$-globulin content and the polarographic indices were not found when percentage composition was considered, indicating that complications by other variable factors may occur. Calculations of the correlation coefficients and inspection of the correlation diagrams shown in Fig. 4 to 6 indicate the absence of relationships between filtrate wave heights or blood indices and $\alpha_2$-, $\beta$-, and $\gamma$-globulin areas.

The general result of the correlation calculations indicates that the increase of blood indices or filtrate wave height values in the pathological group is associated with a component migrating in the $\alpha_1$-globulin fraction of the serum, whilst variation in blood indices and filtrate wave heights in the normal group is not related to any variation of the $\alpha_1$-globulin level. This fact, illustrated in the correlation diagram (Fig. 3), appears to indicate that a new component is present in the pathological sera. At the present stage there is only an indication that this is so, and analytical investigations on the material isolated from pathological and normal sources must be carried out to establish whether it is newly formed or is an increase in quantity of a normal component. Either possibility is in accordance with the suggestion made earlier that the anomalous polarographic and electrophoretic patterns may be due to the mucoproteins demonstrated by Winzler and his colleagues. Only a few electrophoretic measurements have been carried out at pH 4·0 in acetate buffer, but the results obtained indicated that increases in the acid mucoprotein migrating anodically at this pH were related to increases in the $\alpha_1$-globulin content, as has been previously observed by Mehl, Golden and Winzler (1949). Further mobility measurements and preparative experiments are in progress in order to study the polarographic and electrophoretic behaviour of the $\alpha_1$-globulin group.

### SUMMARY.

1. The sera from 32 pathological and 15 normal cases have been studied electrophoretically at pH 8·5, and a smaller number at pH 4·0.

TABLE I.—*The Significance of the Differences between the Amounts of the Components of $n_2$ Pathological Sera and of their Polarographic Reaction compared to $n_1$ Normal Sera, Determined by the "t" Test.*

| Component. | Pooled degrees of freedom $n_1+n_2-2$. | Computed value of "t" for $n_1+n_2-2$ degrees of freedom. | Value of P (differences considered significant for values of P <0·05). | Value of "t" at 5 per cent level for $n_1+n_2-2$ degrees of freedom. | Comment. |
|---|---|---|---|---|---|
| Total protein | 45 | 1·21 | 0·23 | 2·01 | Difference not significant. |
| Total globulin | 45 | 1·95 | 0·055 | 2·01 | Slightly increased but just not significant. |
| Albumin area | 45 | 3·12 | <0·01 | 2·01 | Significantly lower. |
| $\alpha_1$-globulin area | 45 | 2·7 | 0·01 | 2·01 | ,, higher. |
| $\alpha_2$- ,, ,, | 45 | 4·4 | <0·01 | 2·01 | ,, ,, |
| $\beta$- ,, ,, | 45 | 1·76 | 0·08 | 2·01 | Not significantly changed. |
| ,, ,, | 45 | 0·28 | 0·78 | 2·01 | ,, ,, |
| Percentage albumin | 45 | 4·12 | <0·01 | 2·01 | Significantly lower. |
| Percentage $\alpha_1$-globulin area | 45 | 2·93 | <0·01 | 2·01 | ,, higher. |
| ,, $\alpha_2$- ,, ,, | 45 | 7·0 | <0·01 | 2·01 | ,, ,, |
| ,, $\beta$- ,, ,, | 45 | 2·07 | 0·045 | 2·01 | Just significantly higher. |
| ,, ,, ,, | 45 | 1·30 | 0·20 | 2·01 | Not significantly higher. |
| Albumin/globulin ratio | 45 | 3·53 | <0·01 | 2·01 | Significantly lower. |
| Protein index | 37 | 3·3 | <0·01 | 2·02 | , higher. |
| Filtrate wave height | 37 | 4·1 | <0·01 | 2·02 | ,, ,, |
| Blood index | 32 | 3·2 | <0·01 | 2·04 | ,, ,, |

TABLE II.—*Protein Content* and Polarographic Behaviour of Representative Samples of Normal and Pathological Sera.*

| Key. | Sight of tumour or condition. | Total protein. | Total globulin. | Albumin. | A/G ratio. | $\alpha_1$. | $\alpha_2$. | $\beta$. | $\gamma$. | Blood index. | Filtrate. |
|---|---|---|---|---|---|---|---|---|---|---|---|
| Vee | Normal group | 30·6 | 13·9 | 16·2 | 1·16 | 1·1 | 2·3 | 5·1 | 5·4 | 1·08N† | 3·50 |
| Unh. | | 26·6 | 13·8 | 16·3 | 1·18 | 1·0 | 3·4 | 5·3 | 4·1 | 1·70N | 5·75 |
| Sho. | | 32·5 | 13·1 | 18·4 | 1·40 | 1·4 | 2·9 | 3·8 | 5·0 | 2·15+ | 7·25 |
| Erg. | | 35·1 | 15·5 | 19·8 | 1·28 | 1·3 | 2·5 | 5·9 | 5·8 | 0·63N | 2·25 |
| Uck. | | 33·6 | 14·8 | 19·5 | 1·32 | 1·5 | 2·8 | 4·6 | 5·9 | 0·83N | 2·9 |
| Ugo. | | 34·1 | 14·7 | 20·0 | 1·35 | 1·3 | 3·0 | 4·9 | 5·5 | 1·29N | 4·5 |
| Awd. | | 32·7 | 12·2 | 19·5 | 1·60 | 1·0 | 2·3 | 4·7 | 4·2 | 0·74N | 2·6 |
| | Mean | 32·2 | 14·0 | 18·4 | 1·33 | 1·2 | 2·7 | 4·9 | 5·1 | 1·20 | 4·1 |
| | | | | *Pathological Group.* | | | | | | | |
| New. | Breast | 28·6 | 15·45 | 13·15 | 0·85 | 1·7 | 3·2 | 5·4 | 5·3 | 3·22+ | 10·75 |
| ,, | Repeat after 12 weeks | 27·2 | 12·2 | 15·0 | 1·21 | 1·3 | 2·0 | 4·1 | 4·8 | 1·63N | 5·60 |
| Cap. | Breast | 29·8 | 15·0 | 14·8 | 0·97 | 2·0 | 3·8 | 4·9 | 4·3 | 1·89+ | 6·4 |
| ,, | Repeat after 10 weeks | 30·4 | 14·6 | 15·8 | 1·09 | 1·6 | 2·8 | 5·7 | 4·5 | 1·08N | 3·75 |
| Hug. | Breast | 29·2 | 12·2 | 17·0 | 1·39 | 0·0 | 3·7 | 5·3 | 3·3 | 2·53+ | 8·50 |
| Row. | ,, | 26·8 | 13·7 | 13·1 | 0·96 | 1·8 | 3·4 | 5·0 | 3·4 | 3·49+ | 11·1 |
| Tho. | ,, | 28·5 | 16·2 | 12·2 | 0·75 | 2·05 | 5·2 | 5·0 | 4·1 | 3·28+ | 10·1 |
| McA. | Vagina | 26·2 | 12·5 | 13·7 | 1·09 | 1·3 | 3·3 | 4·1 | 3·9 | 1·55N | 5·4 |
| She. | Cervix | 26·0 | 12·1 | 13·8 | 1·13 | 1·2 | 3·2 | 4·1 | 3·8 | 2·12+ | 7·2 |
| Sal. | Prostate | 26·8 | 13·5 | 13·3 | 0·99 | 1·7 | 3·2 | 4·5 | 4·1 | 2·42+ | 8·1 |
| Atk. | Tongue | 27·6 | 16·4 | 11·3 | 0·69 | 2·2 | 2·9 | 4·3 | 7·0 | 3·87+ | 11·5 |
| ,, | Repeat after 6 weeks | 28·9 | 15·7 | 13·2 | 0·84 | 2·1 | 2·6 | 5·1 | 6·0 | 5·05+ | 12·1 |
| Dix. | Mouth | 30·3 | 13·1 | 17·2 | 1·32 | 0·9 | 2·2 | 4·7 | 5·3 | 1·06N | 3·9 |
| Lyn. | Larynx | 31·4 | 13·5 | 17·9 | 1·33 | 1·2 | 3·9 | 4·6 | 3·9 | 1·55N | 5·4 |
| Wil. | Nose | 27·2 | 10·6 | 16·6 | 1·57 | 1·3 | 2·6 | 4·0 | 2·7 | 1·13N | 4·0 |
| Bon. | ,, | 27·5 | 14·1 | 13·4 | 0·96 | 1·9 | 2·9 | 4·9 | 4·4 | 3·48+ | 11·5 |
| Del. | Cheek | 26·0 | 11·2 | 14·8 | 1·32 | 1·2 | 3·1 | 4·1 | 2·8 | 3·26+ | 11·5 |
| Hugg. | Neck | 29·4 | 12·5 | 16·9 | 1·35 | 1·8 | 3·9 | 4·2 | 2·6 | 2·78+ | 9·9 |
| Mor. | Adenoma of thyroid | 33·7 | 20·5 | 13·2 | 0·64 | 1·9 | 4·3 | 4·9 | 9·4 | 1·28N | 4·75 |
| Kea. | Peptic ulcer | 24·0 | 13·3 | 10·8 | 0·81 | 1·9 | 3·6 | 4·6 | 3·3 | 4·50+ | 13·6 |
| Col. | Ulcerative colitis | 27·5 | 14·2 | 13·3 | 0·94 | 1·7 | 3·3 | 4·5 | 4·7 | 6·11+ | 14·1 |
| | Mean | 28·2 | 13·9 | 14·3 | 1·05 | 1·6 | 3·3 | 4·7 | 4·5 | 2·73 | 8·5 |

* Protein component content is expressed arbitrarily in terms of areas of the respective peaks in the electrophoretic diagrams for sera diluted 1 : 2 with veronal buffer, $\mu = 0{\cdot}1$, pH = 8·5.

† Positive (+) and negative (N) results in the polarographic tests are determined with reference to the normal arbitrary levels (Butler, 1951), of 1·7 for B.I. and 6·4 for F.

2. Statistically significant increases of $\alpha_1$- and $\alpha_2$-globulins were found in pathological sera. No statistically significant changes were found in the $\beta$- and $\gamma$-globulin levels. The albumin content and the A/G ratio were lowered.

3. Correlations between the serum composition and polarographic factors are discussed, and their relation to the suggested anomalous mucoprotein content in the $\alpha_1$-globulin fraction is indicated.

We wish to thank Dr. D. A. G. Galton for his assistance in procuring samples of blood. The investigation has been supported by grants from the British Empire Cancer Campaign, the Jane Coffin Childs Memorial Fund for Medical Research, the Anna Fuller Fund and the U.S. Public Health Service.

### REFERENCES.

ABRAMS, A., COHEN, P. P., AND MEYER, O. O.—(1949) *J. biol. Chem.*, **181**, 237.
BRDIČKA, R.—(1933) *Coll. Czech. chem. Commun.*, **5**, 112.
BUTLER, L. O.—(1951) *Brit. J. Cancer*, **5**, 225.
HOCH-LIGETTI, C., AND HOCH, H.—(1948) *Biochem. J.*, **43**, 556.
LONGSWORTH, L. G., AND MACINNES.—(1940) *J. Amer. chem. Soc.*, **62**, 705.
MAYER, K.—(1942) *Z. physiol. Chem.*, **275**, 16.
MEHL, J. W., GOLDEN, F., AND WINZLER, R. J.—(1949) *Proc. Soc. exp. Biol., N.Y.*, **72**, 110.
*Idem*, HUMPHREY, J., AND WINZLER, R. Z.—(1949) *Ibid.*, **72**, 106.
MIDES, G. B., ALLING, E. L., AND MORTON, J. J.—(1950) *Cancer*, **3**, 56.
MOORE, D. H., AND WHITE, J. U.—(1948) *Rev. sci. Instrum.*, **19**, 70.
MÜLLER, O. H., AND DAVIS, J. S.—(1947) *Arch. Biochem.*, **15**, 39.
PETERMANN, M. L., AND HOGNESS, K. R.—(1948*a*) *Cancer*, **1**, 100.—(1948*b*) *Ibid.*, **1**, 104.
*Idem*, KARNOFSKY, D. A., AND HOGNESS, K. R.—(1948) *Ibid.*, **1**, 109.
SEIBERT, F. B., SEIBERT, M. V., ATNO, A. J., AND CAMPBELL, H. W.—(1947) *J. clin. Invest.*, **26**, 90.
SVENSSON, H.—(1943) *Ark. Kemi Min. Geol.*, **17**, No. 14.
WEIMER, H. E., MEHL, J. W., AND WINZLER, R. J.—(1950) *J. biol. Chem.*, **185**, 561.
WINZLER, R. J., DEVOR, A. W., AND MEHL, J. W.—(1947) *Fed. Proc.*, **6**, 303.
*Iidem* AND SMYTH, I. M.—(1948) *J. clin. Invest.*, **27**, 609.
WINZLER, R. J., AND SMYTH, I. M.—(1948) *Ibid.*, **27**, 617.